# Association between cigarette smoking and serum alpha klotho levels among US adults over 40-years-old: a cross-sectional study

**DOI:** 10.1038/s41598-023-46698-5

**Published:** 2023-11-09

**Authors:** Rui Du, Xiaoyan Tang, Meihua Jiang, Shengli Qian, Li Yang, Xiaoling Tong, Wei Huang

**Affiliations:** 1 Department of Ultrasound, General Hospital of Central Theater Command, No.627, Wuluo Road, Wuhan, 430070 Hubei China; 2 Department of Cardiology, General Hospital of Central Theater Command, No.627, Wuluo Road, Wuhan, 430070 Hubei China; 3 Department of Nephrology, General Hospital of Central Theater Command, No.627, Wuluo Road, Wuhan, 430070 Hubei China; 4 Department of Out-patient, General Hospital of Central Theater Command, No.627, Wuluo Road, Wuhan, 430070 Hubei China

**Keywords:** Biomarkers, Risk factors

## Abstract

Alpha klotho (α-Klotho) is an anti-aging molecule associated with aging and several diseases. Previous studies have reported inconsistent levels of α-Klotho in smokers. This study aimed to demonstrate serum α-Klotho levels in smokers among the US population. This cross-sectional study recruited 11,559 participants (aged 40–79 years; 48.2% males). All data were collected from the 2007–2016 National Health and Nutrition Examination Survey. The study comprised adults with reliable Klotho and smoking questionnaire results. The relationship between smoking and serum α-klotho levels was assessed using multivariate linear regression models after adjusting for potential confounders. We also performed a stratified analysis of clinically important variables. The mean serum α-klotho level among the 11,559 participants was 843.85 pg/mL. After full adjustment, habitual smoking was significantly associated with decreased serum levels of α-klotho level (*β* = − 34.89; 95% CI − 54.97, − 14.81; *P* = 0.0013) in the total study population. Furthermore, the stratified analysis indicated that the association was insignificant in the 60–79 age group. Quitting smoking was not significantly associated with serum levels of α-klotho as expected (*P* = 0.1148) in the total study population. However, stratified analyses showed a significant inversed association in the male, those with chronic kidney disease, or those with cancer who quit smoking (all *P* < 0.05). Cigarette smoking was inversely associated with serum α-Klotho levels among US adults.

## Introduction

In 2019, there were 1.14 billion people who smoked cigarettes regularly, and smoking is responsible for 7.69 million deaths and 200 million disability-adjusted life-years^1^. Smoking is considered to have a great impact on life expectancy and makes major contribution to the global burden of morbidity and mortality^2^. Smoking is one of the most prevalent risk factors for a range of diseases, including cardiovascular disease (CVD)^3^, chronic obstructive pulmonary disease (COPD)^4^, and cancer^1^. Meanwhile, smoking is also considered to be responsible for aging, which is a normal physiological process but can be accelerated by cigarette consumption^5^.

Klotho is an important aging suppressor protein that emerged in 1997^6^. Alpha klotho (α-Klotho) protein is a Klotho-encoded transmembrane protein that is highly expressed in the kidney^7^. α-klotho has been reported as a hormone with anti-inflammatory properties. It was also thought to play a vital role in the antioxidative process^8^. Differential expression of α-klotho was also discovered in various diseases, including cardiovascular disease^9^, chronic kidney disease (CKD)^10^, diabetes^11^, systemic lupus erythematosus^12^, and some types of cancer^13^. This may suggest α-klotho may have been involved in these diseases mentioned above.

Several single-center studies with small sample sizes have shown controversial results about the association between cigarette smoking and α-klotho levels^14–16^. Previous studies have demonstrated that serum α-klotho levels were significantly higher in heavy smokers^15^ and decreased following smoking cessation^17^. Additionally, another study found that serum α-klotho levels were significantly higher in male smokers than never-smokers and slightly lower in female smokers^14^. Therefore, the purpose of the present study was to investigate in the relationship between cigarette smoking and serum α-klotho levels in a nationally representative sample of US adults.

## Materials and methods

### Study design and participants

The five continuous 2-year cycles of the National Health and Nutrition Examination Survey (NHANES) data between 2007 and 2016 were analyzed for this study. NHANES was conducted in the US by the National Center for Health Statistics of the Centers for Disease Control and Prevention (CDC). NHANES adopts a stratified, multistage, sophisticated probabilistic design to acquire a representative sample of civilians in the US to examine the health and nutritional status of noninstitutionalized citizens. All participant data is a compilation of household interviews, physical examinations in a mobile examination center (MEC), and laboratory testing performed by highly qualified medical experts. More details about NHANES can be found on the website (https://www.cdc.gov/nchs/nhanes/about_nhanes.htm). The NCHS Ethics Review Board authorized the original research, and all participants obtained written informed consent. The current study was deemed exempt by the Institutional Review Board at the author's institution, as the dataset used in the research was deidentified entirely.

**Figure 1:**
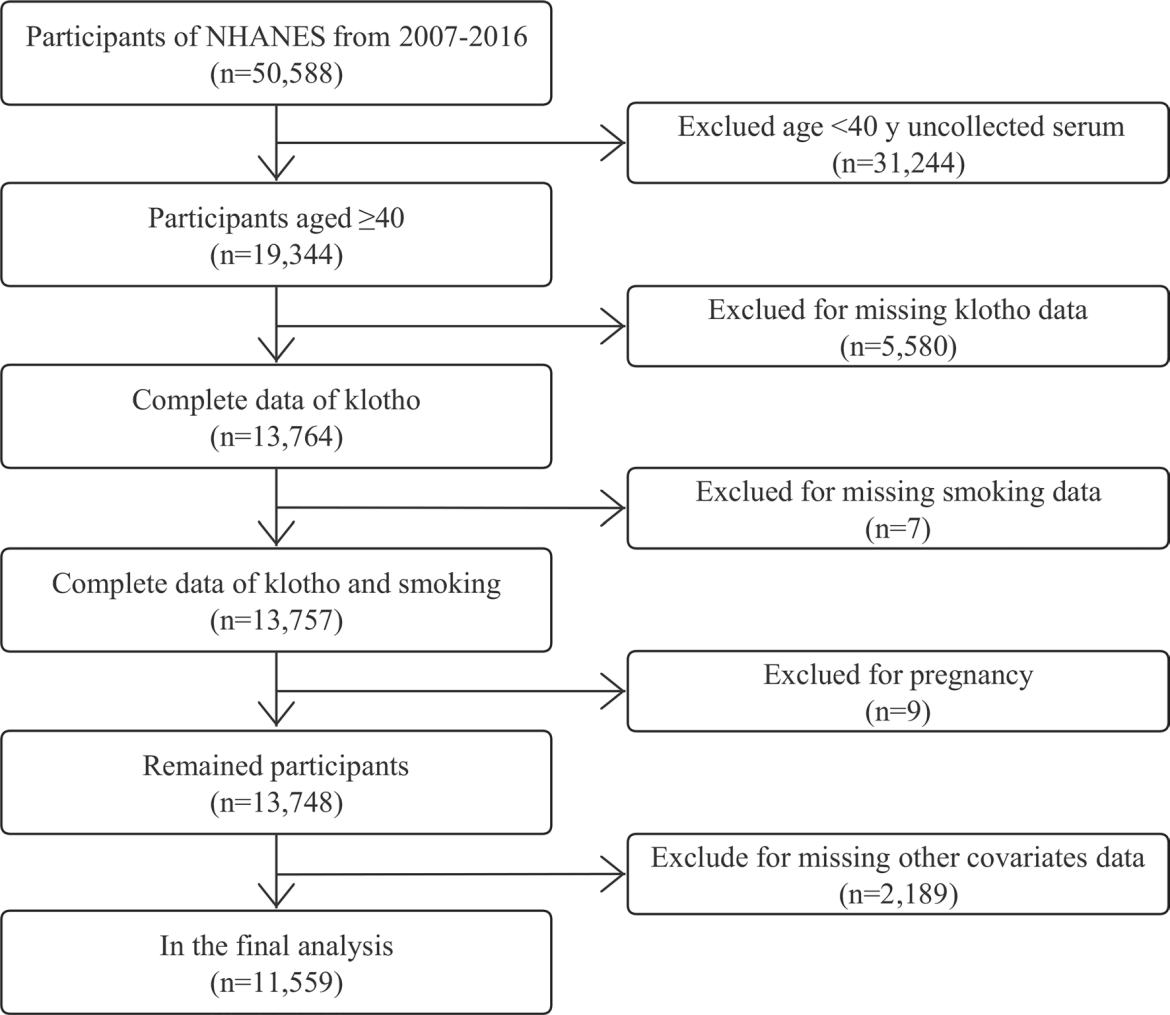
Participants selection flowchart.

We restricted this cross-sectional analysis to participants from the NHANES database from 2007 to 2016 (50,588 individuals in total) because serum α-klotho levels were only tested in NHANES 2007–2016 among participants aged 40–79 who agreed to surplus serum collection for future research. At the time of recruiting, 19,344 were aged 40–79. Of these participants, 5580 were excluded because of missing data on α-klotho, smoking status (n = 7), pregnancy (n = 9), or other covariates (i.e., demographic variables, behavioral factors, history of diseases, n = 2189), resulting in a final analytical sample of 11,559 participants. The flowchart of participant selection was shown in Fig. 1.

### Cigarette smoking

The smoking questions were asked by trained interviewers using the Computer-Assisted Personal Interviewing system at the MEC during the MEC Interview. Survey participants were categorized into 3 groups using self-reported responses for these questionnaires. These are classified as (1) never smoker (less than 100 cigarettes in entire life); (2) quit smoker (100 cigarettes or more in entire life but no smoking now); (3) habitual smoker (smoked 100 cigarettes or more in entire life while smoking now).

### Serum α-klotho level

NHANES Laboratory/Medical Technologists Procedures Manual^18^ contains laboratory methodology and protocol information. Serum α-klotho in frozen serum samples taken during NHANES 2007–2016 were studied in 2019–2020. Fresh-frozen serum samples, stored at -80^◦^C, were measured using an enzyme-linked immunosorbent assay kit manufactured by IBL International, Japan, per the manufacturer's instructions. All samples were analyzed twice, and the average value was calculated. The mean serum α-klotho level was 698.0 pg/mL, ranging from 285.8 to 1638.6 pg/mL^19^.

### Study covariates

The sociodemographic characteristics of each participant, including age, sex, race/ethnicity, marital status, ratio of family income to poverty level, and education level, were self-reported via computer-assisted interviews. Race/ethnicity was collected in five categories: Mexican American, other Hispanic, non-Hispanic White, non-Hispanic Black, and Others. Ratio of family income to poverty (PIR) (< 1.30, 1.30–2.99, and ≥ 3.00)^20^. Education level was divided into (1) Less than high school; (2) High school or GED; (3) Above high school. Body mass index (BMI) was computed using weight and height (kg/m^2^) and classified into the following WHO classes^21^: Normal weight (< 25), Overweight (25–30), and Obese (≥ 30). Alcohol consumption status were collected as a potential confounder classified as^22,23^: (1) never drinker (had < 12 drinks in a lifetime); (2) former drinker (had ≥ 12 drinks in 1 year and did not drink last year, or did not drink last year but drank ≥ 12 drinks in lifetime); (3) light-to-moderate drinker (< 3 drink per day for females, < 4 drinks per day for males on average over the past year, or binge drinking [≥ 4 drinks on same occasion for females, ≥ 5 drinks on same occasion for males] < 5 days per month over the past year); (4) heavy drinker (≥ 3 drinks per day for females, ≥ 4 drinks per day for males on average over the past year, or binge drinking ≥ 5 days per month over the past year). Comorbidities include diabetes, hypertension, CKD, COPD, and CVD. Diabetes was defined as the presence of any of the following conditions: (1) A self-reported diagnosis of diabetes; (2) Use of anti-diabetic drugs; (3) Hemoglobin A1c (HbA1c) level ≥ 6.5%; (4) Fasting plasma glucose level ≥ 7.0 mmol/L; (5) Random plasma glucose level ≥ 11.1 mmol/L. Borderline was defined as (1) Impaired fasting glycemia: Fasting plasma glucose level 6.11–7.0 mmol/L; (2) Impaired glucose tolerance: Random plasma glucose level 7.7–11.1 mmol/L. Hypertension was defined as systolic blood pressure (SBP) value ≥ 140 mmHg and/or diastolic blood pressure (DBP) ≥ 90 mmHg or habitually taking antihypertensive drugs^24^. CKD was defined according to the KDIGO 2021 Clinical Practice Guideline for the Management of Glomerular Diseases^25^. COPD was defined if any of the following: (1) FEV1/FVC < 0.7; (2) A self-reported diagnosis of emphysema; (3) Age above 40 with smoke history or chronic bronchitis and use of phosphodiesterase-4 inhibitors, mast cell stabilizers, leukotriene modifiers, or inhaled corticosteroids. The presence of CVD (including congestive heart failure, coronary heart disease, angina pectoris, heart attack, and stroke) and cancer depended on whether a doctor had told participants they had such disease.

### Statistical analysis

Data was acquired from the NHANES project's nhanesR (http://ckr123.synology.me:3838/nhanesR/). EmpowerStats (http://www.empowerstats.com/cn/, X&Y solutions, Inc., Boston, MA) and R software (The R Foundation; http://www.r-project.org; version 4.2.0) were used for all analyses. *P* < 0.05 is regarded as statistically significant. Strata, primary sampling units, and sample adult weights were used in studies to account for the National Health Interview Survey's complicated design. Weighted linear regression and weighted chi-square test were used to compare baseline characteristics for continuous and categorical variables, respectively. Finally, data were expressed as weighted proportions [95% Confidence interval (CI)] for categorical variables and as weighted means ± Standard Error (SE) for continuous variables. Multivariate linear regression models were set up to test the relationship between smoking and serum α-klotho levels. Following the recommendations for Strengthening the Reporting of Observational Studies in Epidemiology (STROBE), tested four models: Model 1 had no adjustment. Model 2 minimally adjusted for some demographic factors (age, sex, and race/ethnicity). Model 3 additionally adjusted for lifestyle variables (BMI, marital status, PIR, education level, and alcohol consumption status). Model 4 additionally adjusted for clinical variables (diabetes, hypertension, CKD, CVD, COPD, and cancer). Furthermore, stratified subgroup and interaction analyses were performed according to age (< / ≥ 60 years), sex, hypertension, CKD, CVD, and cancer using multivariate linear regression with full adjustment.

### Ethical approval

The ethical approval to conduct the NHANES 2007–2016 was granted by the NHANES Institutional Review Board. The study procedures were structured in line with the Declaration of Helsinki. All participants provided informed consent. The patients/participants provided their written informed consent to participate in this study.

## Results

### Population characteristics

Among 11,559 participants (mean age, 56.2 years; 48.2% male), the mean level of serum α-klotho was 843.85 ± 5.34 pg/mL. The baseline characteristics were presented in Table 1 according to cigarette smoking status. Among all participants, 51.2% have never smoked, 30.4% have quitted smoked, and 18.4% have habitually smoked.

**Table 1:** Baseline characteristic of the participants according to cigarette smoking status.

	Smoking status			*P*-value
	Never smoker (N = 5832)	Quit smoker (N = 3451)	Habitual smoker (N = 2276)	
Age, years	55.4 ± 0.2	59.2 ± 0.3	53.5 ± 0.2	< 0.0001
α-klotho, pg/ml	862.43 ± 6.78	827.67 ± 7.27	818.90 ± 8.94	< 0.0001
Sex (%)				< 0.0001
Female	57.50 (55.98, 59.01)	44.25 (41.69, 46.83)	48.46 (45.89, 51.04)	
Male	42.50 (40.99, 44.02)	55.75 (53.17, 58.31)	51.54 (48.96, 54.11)	
Race/ethnicity (%)				< 0.0001
Mexican American	6.93 (5.39, 8.88)	5.45 (4.27, 6.93)	5.36 (4.00, 7.13)	
Other Hispanic	4.70 (3.70, 5.96)	3.77 (2.86, 4.95)	3.74 (2.74, 5.10)	
Non-Hispanic White	72.71 (69.32, 75.85)	80.12 (77.06, 82.87)	71.92 (67.56, 75.90)	
Non-Hispanic Black	9.06 (7.67, 10.67)	6.35 (5.19, 7.76)	12.22 (10.08, 14.74)	
Others	6.60 (5.57, 7.80)	4.31 (3.48, 5.33)	6.76 (5.17, 8.78)	
BMI (%)				< 0.0001
Normal weight	23.77 (22.18, 25.44)	19.19 (17.12, 21.44)	34.30 (31.75, 36.94)	
Over weight	35.80 (34.08, 37.56)	35.56 (33.31, 37.88)	32.27 (30.10, 34.52)	
Obese	40.43 (38.32, 42.58)	45.25 (42.65, 47.87)	33.43 (30.68, 36.29)	
Marital status (%)				< 0.0001
Married/living with partner	73.52 (71.71, 75.26)	71.91 (69.77, 73.94)	59.12 (56.13, 62.03)	
Living alone	26.48 (24.74, 28.29)	28.09 (26.06, 30.23)	40.88 (37.97, 43.87)	
PIR (%)				< 0.0001
Low	13.86 (12.29, 15.61)	13.72 (11.99, 15.65)	32.02 (28.52, 35.72)	
Middle	23.72 (21.80, 25.76)	27.55 (24.96, 30.31)	30.62 (27.88, 33.51)	
High	62.42 (59.33, 65.40)	58.73 (55.16, 62.20)	37.36 (34.22, 40.61)	
Education level (%)				< 0.0001
Less than high school	11.98 (10.43, 13.73)	15.66 (13.74, 17.81)	25.40 (22.80, 28.19)	
High school or GED	19.24 (17.66, 20.93)	22.29 (20.05, 24.71)	30.66 (27.82, 33.64)	
Above high school	68.77 (66.13, 71.30)	62.04 (58.89, 65.10)	43.94 (40.40, 47.54)	
Alcohol consumption (%)				< 0.0001
Never drinker	17.05 (15.12, 19.17)	3.01 (2.46, 3.69)	2.77 (2.16, 3.55)	
Former drinker	13.73 (12.59, 14.95)	22.87 (20.78, 25.11)	21.25 (19.33, 23.30)	
Light-to-moderate drinker	59.37 (56.58, 62.10)	60.46 (57.50, 63.34)	42.01 (39.64, 44.40)	
Heavy drinker	9.85 (8.92, 10.87)	13.66 (12.06, 15.44)	33.97 (31.53, 36.51)	
Diabetes (%)				< 0.0001
No	72.66 (70.79, 74.45)	64.03 (61.73, 66.27)	73.42 (71.08, 75.65)	
Borderline	9.51 (8.50, 10.63)	12.54 (10.99, 14.26)	9.46 (8.01, 11.14)	
Yes	17.83 (16.39, 19.37)	23.43 (21.24, 25.78)	17.12 (15.39, 19.00)	
Hypertension (%)				< 0.0001
No	54.41 (52.44, 56.38)	45.28 (42.80, 47.78)	52.16 (49.58, 54.74)	
Yes	45.59 (43.62, 47.56)	54.72 (52.22, 57.20)	47.84 (45.26, 50.42)	
CKD (%)				< 0.0001
No	85.44 (84.02, 86.76)	80.65 (78.85, 82.32)	83.70 (82.13, 85.17)	
Yes	14.56 (13.24, 15.98)	19.35 (17.68, 21.15)	16.30 (14.83, 17.87)	
CVD (%)				< 0.0001
No	92.59 (91.72, 93.38)	85.23 (83.73, 86.62)	84.89 (82.80, 86.76)	
Yes	7.41 (6.62, 8.28)	14.77 (13.38, 16.27)	15.11 (13.24, 17.20)	
COPD (%)				< 0.0001
No	97.36 (96.73, 97.87)	88.95 (87.55, 90.20)	84.40 (82.42, 86.19)	
Yes	2.64 (2.13, 3.27)	11.05 (9.80, 12.45)	15.60 (13.81, 17.58)	
Cancer (%)				< 0.0001
No	88.10 (87.08, 89.05)	81.95 (80.07, 83.70)	87.68 (85.60, 89.50)	
Yes	11.90 (10.95, 12.92)	18.05 (16.30, 19.93)	12.32 (10.50, 14.40)	

Mean ± SE for continuous variables; Percentages for categorical variables. All estimates accounted for complex survey designs.

BMI, body mass index; PIR, Ratio of family income to poverty; CKD, chronic kidney disease; CVD, cardiovascular disease; COPD, chronic obstructive pulmonary disease.

Briefly, participants in the quit smoking group were older, more likely to be male, Non-Hispanic White, lived alone, had lower α-klotho levels, and were poorer educated than those in the never smoking group. In addition, quit smokers seemed had higher BMI, more alcohol consumption, and comorbidities. Compared to the never smoking group, participants in the habitual smoking group presented lower serum α-klotho levels. We also noticed that the habitual smoking group was younger, most were male, Non-Hispanic Black, lived alone, and were poorer educated than those in the never smoking group. In addition, the habitual smoking group showed lower BMI, more alcohol consumption, and comorbidities (Table 1).

### Univariate analysis of serum α-klotho levels

The univariate analysis of serum α-klotho levels varied in smoking status, age, sex, race/ethnicity, BMI, alcohol consumption, hypertension, CKD, CVD, and cancer (all *P* < 0.01, Supplementary Table 1).

### Association between smoking and serum α-klotho levels

Four models were conducted to assess the association between smoking and serum α-klotho level, as shown in Table 2. Compared to the never smoking group, the serum α-klotho level was significantly decreased in the habitual smoking group (model 1, *β* = − 43.53; 95% CI − 62.79, − 24.27; *P* < 0.0001). Interestingly, significantly decreased serum α-klotho level was still observed in the population who has quit smoking (model 1, *β* = − 34.76; 95% CI − 50.02, − 19.49; *P* < 0.0001), the trend was significant among the different smoking groups (*P* < 0.0001), which may indicate a negative correlation between serum α-klotho levels and smoking habit. A significantly negative correlation in the habitual smoking group was still observed after adjustment in model 2 (age, sex, race/ethnicity; *β* = − 46.59; 95% CI − 66.37, − 26.80; *P* < 0.0001), model 3 (Further adjusted for BMI, marital status, PIR, education level, alcohol consumption; *β* = − 35.30; 95% CI − 56.75, − 13.85; *P* = 0.002), and model 4 (all covariates; *β* = − 34.89; 95% CI − 54.97, − 14.81; *P* = 0.0013). In the quit smoking group, the decreased serum α-klotho level was still significant in model 2 (*β* = − 19.73; 95% CI − 34.43, − 5.03; *P* = 0.0104). In model 3 (*β* = − 12.16; 95% CI − 27.99, 3.68; *P* = 0.1377) and model 4 (*β* = − 12.54; 95% CI − 27.86, 2.79; *P* = 0.1148), the serum α-klotho level was decreased without statistical significance. The trend remained significant among the different smoking groups in model 4 (*P* = 0.0011).

**Table 2:** Associations between smoking and serum α-klotho level.

	Model 1		Model 2		Model 3		Model 4	
	β (95% CI)	*P*-value	β (95% CI)	*P*-value	β (95% CI)	*P*-value	β (95% CI)	*P*-value
Never smoking	Ref		Ref		Ref		Ref	
Quit smoking	− 34.76 (− 50.02 to − 19.49)	< 0.0001	− 19.73 (− 34.43 to − 5.03)	0.0104	− 12.16 (− 27.99 to 3.68)	0.1377	− 12.54 (− 27.86 to 2.79)	0.1148
Habitual smoking	− 43.53 (− 62.79 to − 24.27)	< 0.0001	− 46.59 (− 66.37 to − 26.80)	< 0.0001	− 35.30 (− 56.75 to − 13.85)	0.002	− 34.89 (− 54.97 to − 14.81)	0.0013
*P* for trend		< 0.0001		< 0.0001		0.0021		0.0011

Model 1: crude model.

Model 2: adjusted for age + sex + race/ethnicity.

Model 3: adjusted for Model 2 + BMI + marital status + PIR + education level + alcohol consumption.

Model 4: adjusted for Model 3 + diabetes + hypertension + CKD + CVD + COPD + cancer.

95%CI, 95% Confidence interval; BMI, body mass index; PIR, Ratio of family income to poverty; CKD, chronic kidney disease; CVD, cardiovascular disease; COPD, chronic obstructive pulmonary disease.

### Subgroup analyses

**Figure 2:**
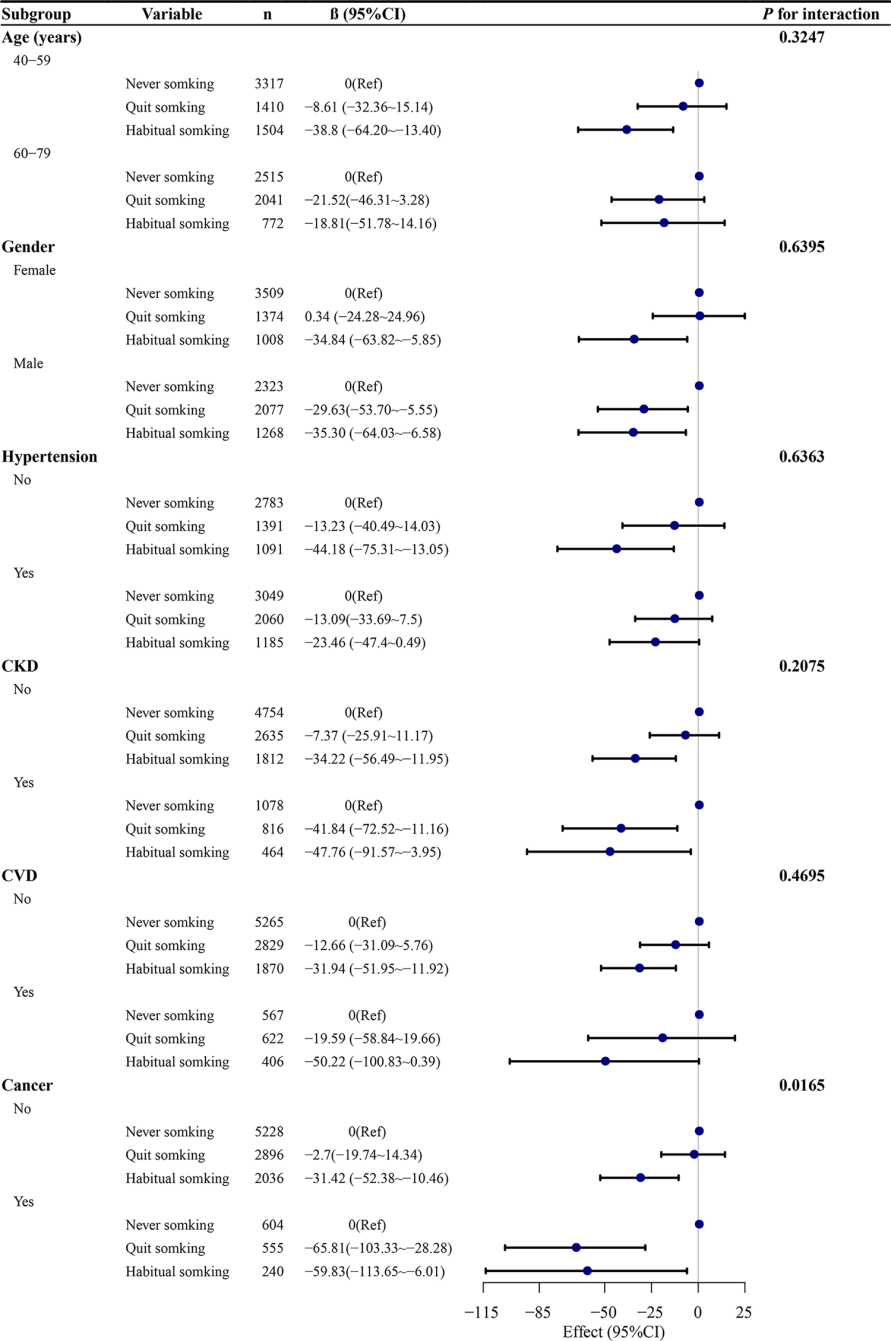
Subgroup analyses of the association between smoking and serum α-Klotho level (pg/mL). Each stratification adjusted for all factors (age, sex, race/ethnicity, BMI, marital status, PIR, education level, alcohol consumption, diabetes, hypertension, CKD, CVD, COPD, and cancer) except the stratification factor itself. BMI, body mass index; PIR, Ratio of family income to poverty; CKD, chronic kidney disease; CVD, cardiovascular disease; COPD, chronic obstructive pulmonary disease.

In age, sex, hypertension, CKD, CVD, and cancer subgroup, most subgroups showed the serum α-klotho levels were significantly decreased in the habitual smoking group but not in the quit smoking group (Fig. 2). However, no significant correlation was observed in the habitual smoking group aged 60–79 years old, those with hypertension or CVD (all *P* > 0.05). Serum α-Klotho levels were more significantly reduced in male, participants with CKD or cancer in the quit smoking groups than in the never smoking groups (all *P* < 0.05). We found evidence of a significant interaction between cancer and smoking exposure (*P* for interaction = 0.0165).

## Discussion

Smoking is considered contributed to the shortening of life expectancy. Our study showed that the serum level of α-klotho, a potential aging biomarker, was negatively correlated with cigarette smoking.

Numerous studies have supported the association between smoking and α-Klotho levels. A previous study has shown that the serum levels of soluble a-klotho were significantly higher in heavy smokers among 40 smokers (40–60 years, ≥ 6 pack cigarette per year) without COPD^15^. In another study that enrolled 230 health participants (90 men and 140 women), the serum α-Klotho level was significantly higher in smokers than in never-smokers in males. However, the serum α-Klotho level was slightly lower in the female smokers^14^. Interestingly, α-Klotho levels decreased for smoking cessation (n = 28)^17^. However, most of these studies are based on small samples, and the results are inconsistent^14,15,17,26^. In our research, we found the serum α-Klotho level was significantly lower in habitual smokers than in never smokers, which was consistent with a previous study^26^.

Our study found no significant relationship between habitual smoking and levels of α-Klotho in individuals over 60 years old. It is important to mention that α-Klotho is acknowledged as an anti-aging protein, and its serum levels tend to diminish as individuals progress in age^27^. The findings of our study align with previous report^28^, which could explain why significant results were observed only in the 40–59 age group. In the male, CKD and cancer population, smoking cessation is related to lowering the serum α-Klotho. These results may not indicate a protective role of smoking cessation in these subgroups as expected. However, previous studies^29,30^ showed that the α-Klotho level was more significantly correlated with the severity of these diseases than smoking. Furthermore, it is worth noting that the current study did not consider the duration of smoking cessation, which is an important factor in assessing the dose–response relationship. The time since quitting smoking can significantly influence the impact on α-Klotho levels^17^. Therefore, future research should consider examining the relationship between smoking cessation duration and α-Klotho levels to provide a more comprehensive understanding of the effects of quitting smoking on this biomarker.

These findings may indicate some association between smoking and aging. Cellular senescence includes inflammation, fibrosis, metabolic disorders, DNA damage, mitochondrial dysfunction, loss of protein homeostasis, failed autophagy, Reactive Oxygen Species generation, NAD + depletion, and stem/progenitor cell dysfunction^31–33^. A mixture of thousands of chemicals is created by burning or heating tobacco when people smoke. Reactive oxygen species (ROS), one of these chemicals, can activate epithelial intracellular cell signaling cascades and lead to inflammatory gene activation. ^34^ The downstream of the inflammatory cascades including the Tumor necrosis factor (TNF), interferon γ (IFN- γ), and interleukin-6 (IL-6)^34,35^ can downregulate the α-klotho gene expression^36^. Oxidative stress has been shown to decrease the expression of the α-Klotho gene^37^. Smoking-induced oxidative stress was also observed in humans, animal models, and in vitro cell culture systems^38,39^. α-Klotho deficiency was also directly or indirectly instrumental in lowering autophagic flux by causing phosphotoxicity^40,41^. Cigarette smoke exposure induces ROS-mediated autophagy by regulating sestrin, AMPK, and mTOR levels^42,43^. All the above molecules (ROS, TNF, IFN- γ, IL-6, oxidative stress, AMPK, and mTOR) were closely related to the biological process of aging^44–46^.

We revealed lower serum α-klotho in smokers which may suggest smoking may accelerate aging by decreasing the anti-aging factor klotho. This study’s large sample size and use of a representative multiethnic population allows for better generalization to the US population. Further studies are required to prove this hypothesis.

Nevertheless, the present study has several limitations. First, a bias may exist for the self-reporting smoking status, which may be discrepancy with the real smoking status in the database. Second, we can only observe the correlation between smoking and α-klotho but not affirm the causal relationship between the two factors in an observational study. Third, we can't acquire complete information on the cigarette type and accurate dose of cigarette consumption in the NHANES database, which may prevent a further study between cigarette consumption and α-Klotho levels. Additionally, it is important to acknowledge that the current study did not investigate the time dose–response relationship in relation to smoking cessation, which could potentially influence the findings.

## Conclusions

In conclusion, we proved habitual smoking is associated with a lower serum level of α-Klotho, which was considered an anti-aging factor. The result may indicate that smoking may accelerate aging by affecting α-Klotho levels. Further studies are required to confirm these results and convince the underlying mechanism.

### Supplementary Information

Supplementary Table 1.

## Data Availability

Some or all datasets generated during and/or analyzed during the current study are not publicly available but are available from the corresponding author on reasonable request.
