# Supplementary material for: Association between cigarette smoking and serum alpha klotho levels among US adults over 40-years-old: a cross-sectional study
Source: Sci Rep. 2023 Nov 9;13:19519. doi: 10.1038/s41598-023-46698-5 (PMC10636022; doi:10.1038/s41598-023-46698-5)
Supplement: Supplementary file 1 — Supplementary Table 1. [file 41598_2023_46698_MOESM1_ESM.docx]

**Supplementary Table 1. Univariate analysis for α-klotho level (pg/ml)**

|  | **N** | **Mean (95%CI)** | **β (95%CI)** | ***P*** |
| --- | --- | --- | --- | --- |
| **Smoking status** |  |  |  |  |
| Never smoker | 5832 | 862.43 (849.14, 875.71) | Ref. |  |
| Quit smoker | 3451 | 827.67 (813.42, 841.92) | -34.76 (-50.02, -19.49) | <0.0001 |
| Habitual smoker | 2276 | 818.90 (801.38, 836.42) | -43.53 (-62.79, -24.27) | <0.0001 |
| **Age** | 11559 | 843.85 (833.39, 854.31) | -2.20 (-2.83, -1.57) | <0.0001 |
| **Sex** |  |  |  |  |
| Female | 5891 | 861.11 (848.20, 874.02) | Ref. |  |
| Male | 5668 | 825.31 (813.00, 837.61) | -35.80 (-49.86, -21.74) | <0.0001 |
| **Race/ethnicity** |  |  |  |  |
| Mexican American | 1768 | 852.68 (833.89, 871.47) | Ref. |  |
| Other Hispanic | 1247 | 873.51 (848.40, 898.62) | 20.83 (-8.59, 50.25) | 0.1693 |
| Non-Hispanic White | 5242 | 833.74 (822.29, 845.19) | -18.94 (-39.87, 2.00) | 0.0803 |
| Non-Hispanic Black | 2282 | 907.55 (882.56, 932.54) | 54.87 (24.43, 85.31) | 0.0007 |
| Others | 1020 | 846.30 (821.06, 871.53) | -6.39 (-34.12, 21.35) | 0.6531 |
| **BMI** |  |  |  |  |
| Normal weight | 2696 | 867.96 (849.25, 886.66) | Ref. |  |
| Over weight | 4001 | 836.45 (820.51, 852.40) | -31.51 (-54.04, -8.98) | 0.0076 |
| Obese | 4862 | 835.82 (824.37, 847.28) | -32.13 (-51.73, -12.54) | 0.0019 |
| **Marital status** |  |  |  |  |
| Married/living with partner | 7474 | 839.48 (827.74, 851.21) | Ref. |  |
| Living alone | 4085 | 854.26 (839.94, 868.58) | 14.79 (-0.62, 30.20) | 0.0638 |
| **PIR** |  |  |  |  |
| <1.30 | 3458 | 846.66 (832.49, 860.83) | Ref. |  |
| 1.30−2.99 | 3511 | 840.41 (824.35, 856.47) | -6.25 (-24.68, 12.18) | 0.5082 |
| ≥3.00 | 4590 | 844.59 (831.52, 857.67) | -2.07 (-19.33, 15.19) | 0.8147 |
| **Education level** |  |  |  |  |
| Less than high school | 3068 | 844.57 (825.97, 863.18) | Ref. |  |
| High school or GED | 2584 | 822.66 (807.99, 837.32) | -21.92 (-45.15, 1.31) | 0.0683 |
| Above high school | 5907 | 851.27 (837.39, 865.15) | 6.69 (-14.42, 27.81) | 0.5363 |
| **Alcohol consumption** |  |  |  |  |
| Never drinker | 1631 | 884.57 (862.12, 907.03) | Ref. |  |
| Former drinker | 2508 | 848.21 (833.49, 862.93) | -36.37 (-62.45, -10.29) | 0.0078 |
| Light-to-moderate drinker | 5653 | 846.28 (833.58, 858.99) | -38.29 (-60.76, -15.82) | 0.0013 |
| Heavy drinker | 1767 | 803.14 (784.81, 821.46) | -81.44 (-109.25, -53.62) | <0.0001 |
| **Diabetes** |  |  |  |  |
| No | 7388 | 843.90 (832.29, 855.51) | Ref. |  |
| Borderline | 1201 | 840.18 (816.59, 863.77) | -3.72 (-27.68, 20.24) | 0.7617 |
| Yes | 2970 | 845.67 (828.93, 862.41) | 1.77 (-15.37, 18.91) | 0.8403 |
| **Hypertension** |  |  |  |  |
| No | 5265 | 853.82 (842.27, 865.37) | Ref. |  |
| Yes | 6294 | 833.39 (820.63, 846.14) | -20.43 (-32.76, -8.10) | 0.0017 |
| **CKD** |  |  |  |  |
| No | 9201 | 851.91 (840.40, 863.42) | Ref. |  |
| Yes | 2358 | 802.60 (786.71, 818.49) | -49.31 (-66.99, -31.63) | <0.0001 |
| **CVD** |  |  |  |  |
| No | 9964 | 848.18 (837.34, 859.01) | Ref. |  |
| Yes | 1595 | 809.11 (789.62, 828.59) | -39.07 (-58.30, -19.84) | 0.0002 |
| **COPD** |  |  |  |  |
| No | 10683 | 845.15 (834.32, 855.97) | Ref. |  |
| Yes | 876 | 828.10 (802.21, 853.98) | -17.05 (-43.72, 9.62) | 0.2140 |
| **Cancer** |  |  |  |  |
| No | 10160 | 848.63 (838.17, 859.09) | Ref. |  |
| Yes | 1399 | 814.13 (795.75, 832.51) | -34.50 (-51.04, -17.97) | 0.0001 |

Note: 95%CI, 95% Confidence interval; Mean: survey-weighted mean

**Abbreviations:** BMI, body mass index; PIR, Ratio of family income to poverty; CKD, chronic kidney disease; CVD, cardiovascular disease; COPD, chronic obstructive pulmonary disease.
